# Supplementary material for: Flavonoid–Phenolic Acid Hybrids Are Potent Inhibitors of Ferroptosis via Attenuation of Mitochondrial Impairment
Source: Antioxidants (Basel). 2023 Dec 26;13(1):44. doi: 10.3390/antiox13010044 (PMC10812788; doi:10.3390/antiox13010044)
Supplement: Supplementary file 1 [file antioxidants-13-00044-s001.zip › antioxidants-2788124-supplementary.pdf]

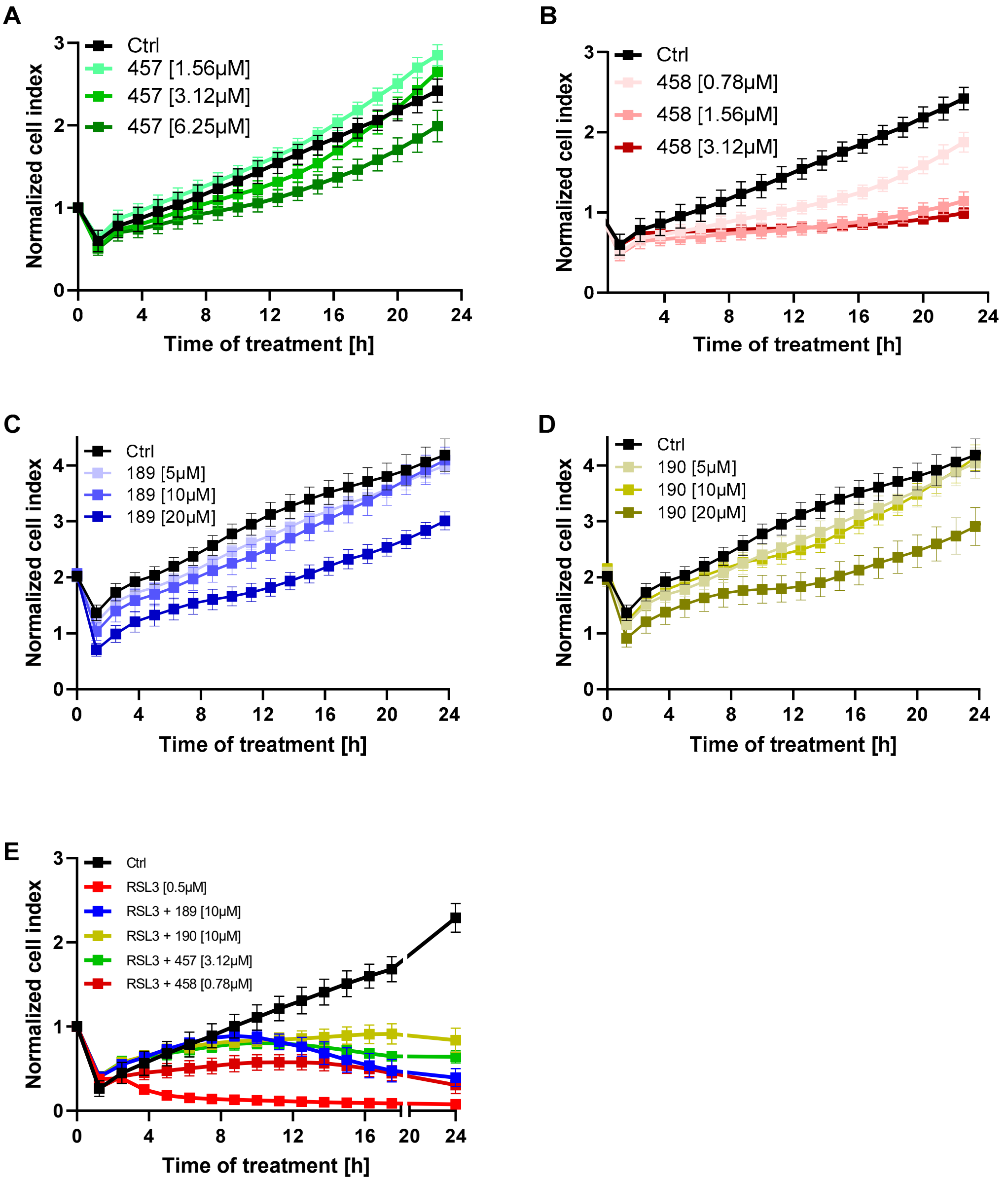

Figure S1. Proliferation monitoring of neuronal HT22 cells in presence of the flavonoid-phenolic acid hybrids. (A-E) Proliferation was assessed via impedance based XCELLigence system. Shown are the normalized proliferation curves from a representative experiment with six replicates per conditions, depicted as mean +/- SD.

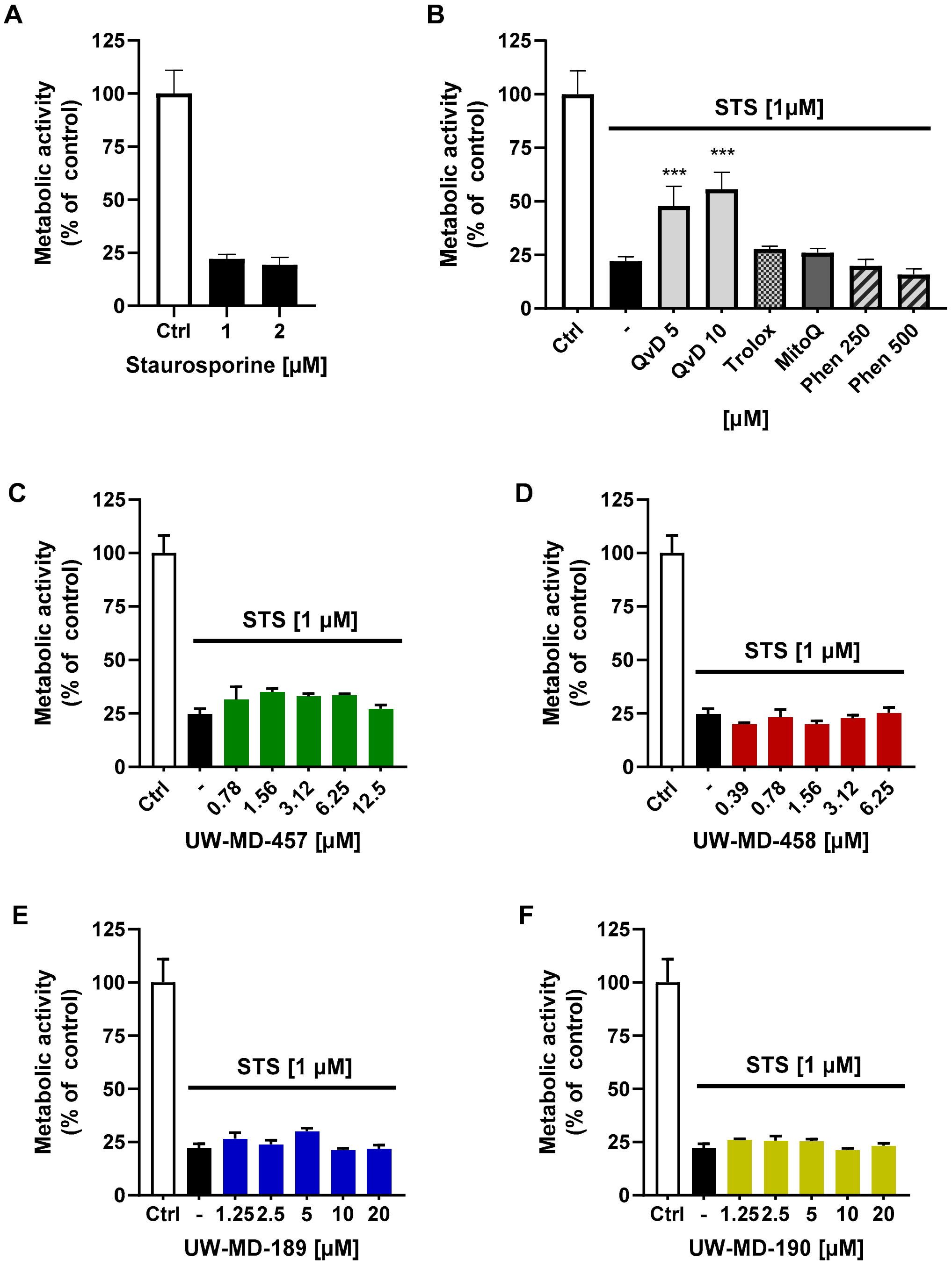

Figure S2. Effects of UW-MD in models of staurosporine-induced apoptotic cell death. (A) HT22 cells were incubated with 1 and 2 µM staurosporine for 24 h. Cell viability was determined by measuring MTT absorbance. **(B)** Pharmacological interference in apoptosis-induced cell death in response to the pan-Caspase inhibitor QvD-OPh (5-10 µM), to the radical scavenger Trolox (50-100µM), the mitochondrial targeted antioxidant MitoQ (0.3 µM), and the mitochondrial complex I inhibitor phenformin (250-500 µM) for 24 h incubation. **(C-F)** Effects of the hybrids in models of apoptosis. HT22 cells were incubated with 1 µM staurosporine and indicated concentrations of UW-MD for 24h. Cell viability was determined using MTT assay. Data represent the means +/- SD of six replicates from one representative experiment.

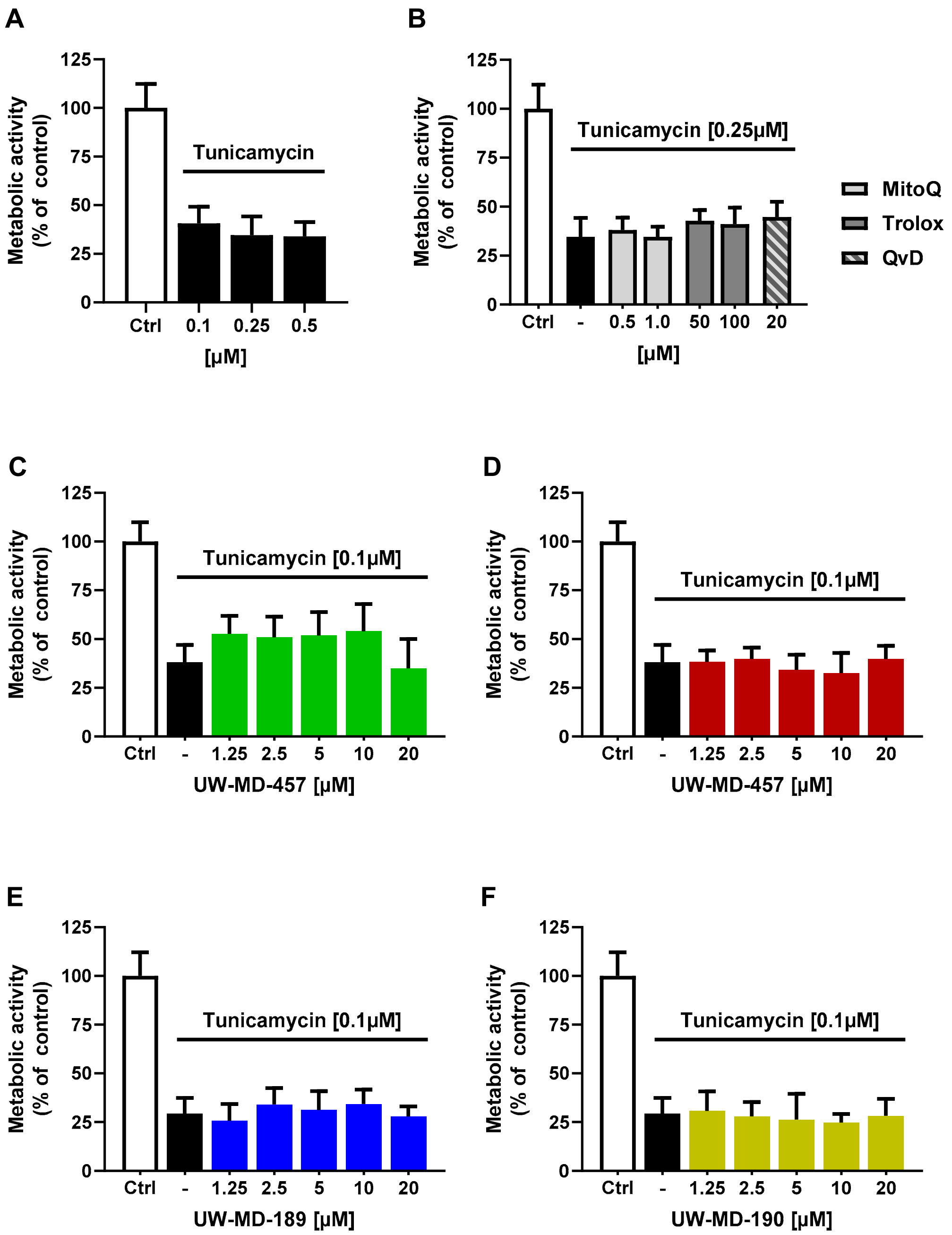

Figure S3. Effects of UW-MD in models of tunicamycin-induced ER-stress. (A) HT22 cells were incubated with various concentrations of tunicamycin for 24 h. Cell death was determined by measuring MTT absorbance. **(B)** Pharmacological interference in tunicamycin-induced ER stress response with the mitochondrial targeted antioxidant MitoQ (0.5-1 µM), the radical scavenger Trolox (50-100µM) and the pan-caspase inhibitor QvD-OPh (20µM) after 24 h incubation. **(C-F)** Effects of the hybrids in models of ER-stress. HT22 cells were incubated with 0.1 µM tunicamycin and indicated concentrations of UW-MD for 24h. Cell viability was determined using MTT assay. Data represent the means +/- SD of six replicates from one representative experiment.

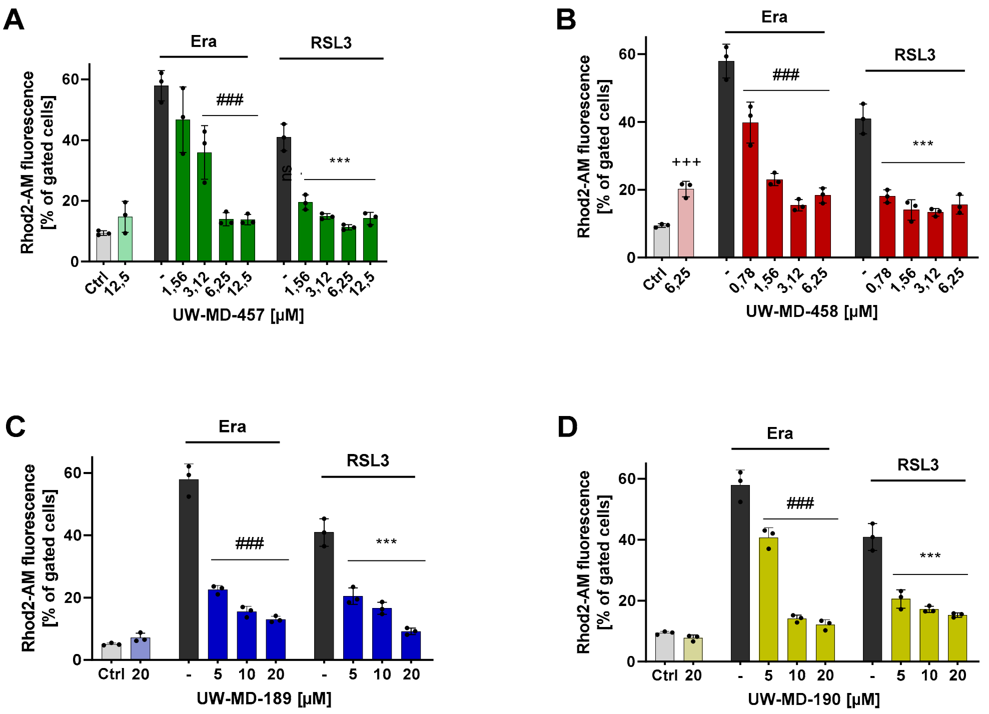

Figure S4. Mitochondrial Ca^{2+} accumulation upon compound treatment in models of oxidative stress. (A-D) Flow cytometry analysis of Rhod-2 flourescence in HT22 cells 8 h after compound co-incubation. Data are presented as mean +/-SD, n=3, +++ $p < 0.001$ compared to untreated control, ### $p < 0.001$ compared to erastin-treated control, *** $p < 0.001$ compared to RSL3-treated control, ANOVA, Bonferroni's test.

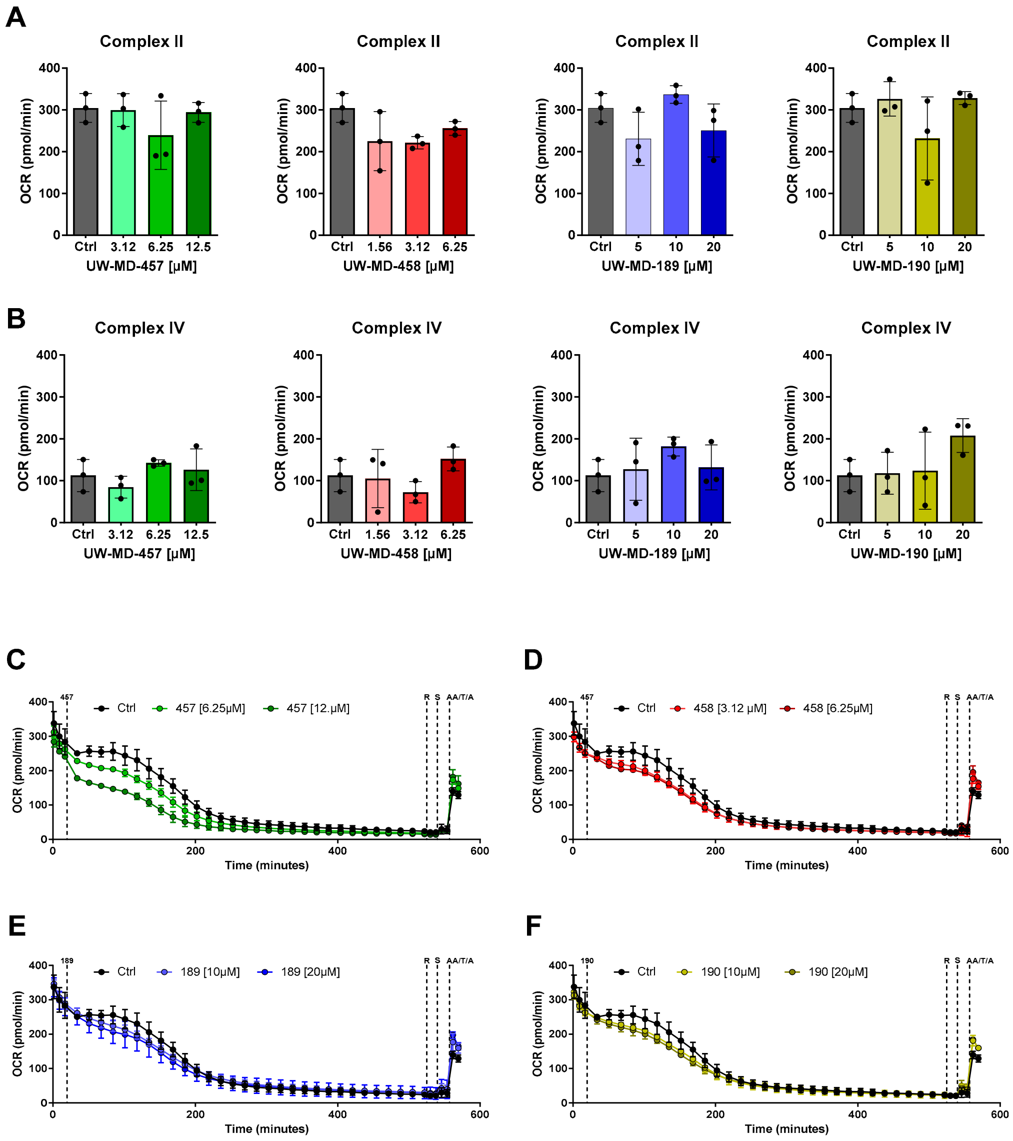

Figure S5. Seahorse electronen flow assay showed no actions of the flavonoid-phenolic acid hybrids on complex II and complex IV acitivity of the electron transport chain. (A) Effects of the hybrid compounds on succinate driven OCR in permeabilized HT22 cells after 3 h pre-incubation. Charts represent calculated complex II activity averaging four technical replicates. **(B)** Quantification of complex IV activity from TMPD/ascorbate-stimulated OCR of permeabilized HT22 cells from four individual replicates. **(C-F)** HT22 cells were individually treated with the hybrids and OCR was monitored in real time. OCR were measured in permeabilized cells supplemented with pyruvate, glutamine and ADP from four replicates per condition.
